# Highly specific targeted mutagenesis in plants using *Staphylococcus aureus* Cas9

**DOI:** 10.1038/srep26871

**Published:** 2016-05-26

**Authors:** Hidetaka Kaya, Masafumi Mikami, Akira Endo, Masaki Endo, Seiichi Toki

**Affiliations:** 1 Plant Genome Engineering Research Unit, Institute of Agrobiological Sciences, National Agriculture and Food Research Organization, 2-1-2 Kannondai, Tsukuba, Ibaraki 305-8602, Japan; 2 Graduate School of Nanobioscience, Yokohama City University, 22-2 Seto, Yokohama, Kanagawa 236 0027, Japan; 3 Kihara Institute for Biological Research, Yokohama City University, 641-12 Maioka-cho, Yokohama, Kanagawa 244-0813, Japan

## Abstract

The CRISPR/Cas9 system is an efficient and convenient tool for genome editing in plants. Cas9 nuclease derived from *Streptococcus pyogenes (Sp*) is commonly used in this system. Recently, *Staphylococcus aureus* Cas9 (SaCas9)-mediated genome editing was reported in human cells and *Arabidopsis*. Because SaCas9 (1053 a.a.) is smaller than SpCas9 (1368 a.a.), SaCas9 could have substantial advantages for delivering and expressing Cas9 protein, especially using virus vectors. Since the protospacer adjacent motif (PAM) sequence of SaCas9 (5′-NNGRRT-3′) differs from that of SpCas9 (5′-NGG-3′), the use of this alternative Cas9 nuclease could expand the selectivity at potential cleavage target sites of the CRISPR/Cas9 system. Here we show that SaCas9 can mutagenize target sequences in tobacco and rice with efficiencies similar to those of SpCas9. We also analyzed the base preference for ‘T’ at the 6th position of the SaCas9 PAM. Targeted mutagenesis efficiencies in target sequences with non-canonical PAMs (5′-NNGRRV-3′) were much lower than those with a canonical PAM (5′-NNGRRT-3′). The length of target sequence recognized by SaCas9 is one or two nucleotides longer than that recognized by SpCas9. Taken together, our results demonstrate that SaCas9 has higher sequence recognition capacity than SpCas9 and is useful for reducing off-target mutations in crop.

The clustered regularly interspaced short palindromic repeat (CRISPR)-associated endonuclease (CRISPR/Cas) system, the feature widely observed in prokaryote genomes, functions as an adaptive immune system by installing short DNA fragments from viruses or plasmids into CRISPR loci using RNA from the CRISPR loci to target specific sequences in invader genomes^1^,^2^. Of the three types of CRISPR/Cas systems, type II has been most intensively studied and developed for genome engineering techniques in eukaryotes, including plants^3^,^4^,^5^,^6^,^7^,^8^,^9^.

The type II CRISPR/Cas system is composed of a Cas9 nuclease and a guide RNA (gRNA)^10^,^11^. The chimeric single gRNA comprises two parts: a scaffold, and a specific DNA target sequence on the 5′ end. The Cas9 nuclease most commonly used in genome editing research to date is derived from *Streptococcus pyogenes (Sp*)^12^. SpCas9 mediates genome editing at a site complementary to a 20-nucleotide DNA specific sequence in the gRNA^13^. Cas9 requires a protospacer adjacent motif (PAM) at the 3′ end of the DNA target sequence to recognize and cleave the target DNA^14^. The PAM sequence for SpCas9 is 5′-NGG-3′^15^.

Several Cas9 protein homologs have been identified and characterized for use in genome editing research^16^. Each Cas9 protein homolog functions similarly as an RNA-guided endonuclease, with homologs differing in their molecular weights and PAM preferences. Recently, Ran *et al*. reported that, when different Cas9 homologues were tested in mammalian cells, *Staphylococcus aureus* Cas9 (SaCas9) exhibited robust activity^17^. SaCas9 (1053 amino acids) is smaller than SpCas9 (1368 amino acids). The relatively large size of SpCas9 has limited its utility, thus the smaller Cas9 from *Staphylococcus aureus* might prove useful for therapeutic applications using the versatile adeno-associated virus delivery vehicle^17^. Unlike SpCas9, SaCas9 requires a 21- or 22-nt DNA specific sequence and the motif 5′-NNGRRT-3′ as a PAM sequence^17^. To broaden the spectrum of genome editing using the CRISPR/Cas9 system in plants, we need to widen the choice of Cas9 proteins beyond SpCas9.

During the preparation of this paper, Steinert *et al*. reported that SaCas9 could induce mutation in *Arabidopsis thaliana*^18^. Here, we show that SaCas9 functions in rice and tobacco. The probability of off-target mutation induced by SaCas9 might be lower than that induced by SpCas9. SaCas9 prefers the canonical PAM for effective targeted mutagenesis. Our results suggest that SaCas9 can direct highly specific genome editing activity, and will be useful for targeted genome editing in crops.

## Results

### Comparing the efficiencies of SaCas9 and SpCas9 for genome editing in tobacco

**Figure 1:**
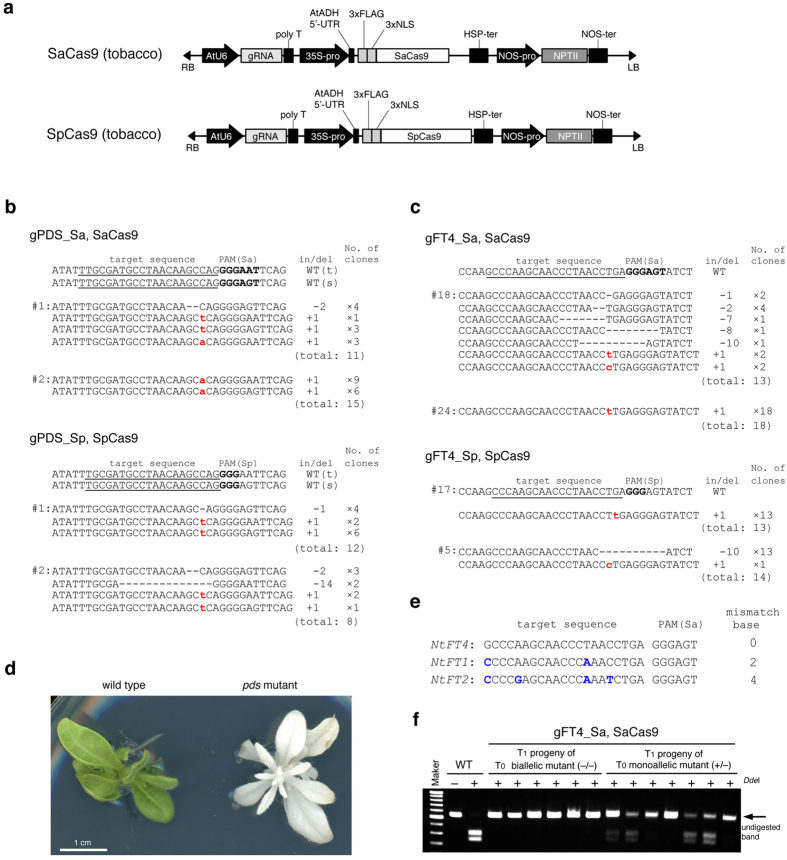
SaCas9 induced mutation in tobacco. (**a**) Expression construct for *SaCas9* (top) and *SpCas9* (bottom) in tobacco. A 3 × FLAG tag and 3 × NLS peptide were translationally fused in tandem to the N-terminus of Cas9. The AtADH 5′-UTR and HSP terminator enhance transcription of the *Cas9* gene. NLS: nuclear localization signal. AtADH 5′-UTR: 5′ untranslated region of *Arabidopsis thaliana ALCOHOL DEHYDROGENASE* gene. HSP-ter: the terminator region of *Arabidopsis thaliana HEAT SHOCK PROTEIN 18.2* gene. (**b**,**c**) Mutations detected in *NtPDS* (**b**) and *NtFT4* (**c**) gene loci. The wild-type sequence is shown at the top with the target sequence underlined and the PAM in bold. DNA deletions and insertions are shown in dashes and lower case red letters. The net change in length, and the number of clones are noted to the right of each sequence (+, insertion; −, deletion; ×, number of clones). (t): Genomic DNA derived from *N. tomentosiformis*, (s): genomic DNA derived from *N. sylvestris*. (**d**) Phenotype of *pds* mutant. *Left* A regenerated plant with wild-type *PDS* gene, *right* a regenerated plant with mutation in all four *NtPDS* genes. (**e**) Potential off-target genes for *NtFT4*. The target sequence of the *NtFT4* gene is shown at the top with PAM. The mismatched bases in *NtFT1* and *NtFT2* genes are shown in blue letters. (**f**) CAPS analysis of *NtFT4* gene locus in the T_1_ generation. Each T_1_ progeny was segregated from biallelic mutant (T_0_) or monoallelic mutant (T_0_). -: Non-digested PCR products, +: *Dde*I-digested PCR products. An undigested band indicates mutation in the *NtFT4* gene.

A synthetic *SpCas9* gene^19^ and the *SaCas9* gene optimized for *Arabidopsis thaliana* codon usage were expressed to induce mutation in the tobacco genome. Expression of both *Cas9* genes was controlled by the Cauliflower mosaic virus (CaMV) 35S promoter (35S) (Fig. 1a). The gRNA, consisting of a sequence specific to the target DNA and a scaffold sequence, was expressed under the control of the *U6-26* promoter^20^ derived from *Arabidopsis thaliana*. We selected two target genes in tobacco (*Nicotiana tabacum*): the *PHYTOENE DESATURASE (NtPDS*) gene^21^ and the *NtFT4* gene^22^. Tobacco is an allotetraploid derived from ancestors of two diploids, *Nicotiana tomentosiformis* and *Nicotiana sylvestris*, thus there are two homologous genes for *PDS* and four homologous genes (*FT1*–*4*) for *FT* in the tobacco genome. The *PDS* gene encodes a phytoene desaturase involving in carotenoid biosynthesis and disruption of *PDS* causes albino phenotype resulting from lack of carotenoid derivatives such as chlorophyll^23^. The *NtFT4* gene induces flowering in tobacco, while the other three (*NtFT1–3*) repress flowering^22^. To evaluate the genome editing activity of SaCas9 in comparison with that of SpCas9, we selected target sequences of 21 nt and 20 nt, the 3′ end of which have 5′-NNGRRT-3′ and 5′-NGG-3′ as the PAM sequences for SaCas9 and SpCas9, respectively (Table 1). To eliminate position effects on genome editing, the two target sequences (SaCas9 and SpCas9) were designed to be mostly same (Table 1). Transgenic tobacco was produced by Agrobacterium-mediated transformation with the constructs shown in Fig. 1a. The transformed tobacco (T_0_ generation) was regenerated from leaf disks on kanamycin plates for 4–6 weeks (Supplementary Fig. S1a). Genomic DNA was extracted independently from each regenerated shoot and subjected to cleaved amplified polymorphic sequences (CAPS) analysis to assess the presence or absence of mutations in the target sequences. The genome editing frequency was estimated by determining the ratio of regenerated shoots with mutation to those without mutation. The mutation frequency induced by SaCas9 at the *NtPDS* and *NtFT4* gene loci was 75.6% and 65.1%, respectively (Table 2). These results indicate that SaCas9 can induce targeted mutation in tobacco. The mutation frequency induced by SaCas9 was almost same as that induced by SpCas9 at both loci (Table 2). We examined the patterns of mutation induced by SaCas9 and SpCas9 at the *NtPDS* gene and *NtFT4* gene loci (Fig. 1b,c). SaCas9 most often induced small deletions or small insertions around the PAM sequence (Fig. 1b,c). Some transgenic tobacco plants expressing *gPDS_Sa* and *SaCas9* showed an albino phenotype; plants with this phenotype possessed biallelic mutation in two *NtPDS* genes (Fig. 1d).

### Analysis of off-target mutations induced by SaCas9

We examined the possibility that SaCas9 induces off-target mutations in tobacco. The *NtFT1* and *NtFT2* genes have two and four mismatched bases, respectively, in the *NtFT4* target sequence (Fig. 1e). No regenerated plants with a mutation in the *NtFT1* or *NtFT2* gene were detected, suggesting that SaCas9 does not induce mutation in a target sequence that has two mismatched bases in the corresponding gRNA sequence (Table 3).

### Heritable targeted mutagenesis using SaCas9

We next examined whether the mutation induced by SaCas9 is inherited by the next generation. Because the *pds* null mutant is seedling lethal, we analyzed the mutation in the *NtFT4* gene. Genomic DNA extracted independently from the T_1_ progenies of biallelic (T_0_) and monoallelic (T_0_) mutant was subjected to CAPS analysis. PCR products that were not digested by restriction enzyme (*Dde*I) indicated that the mutation had been introduced into *NtFT4* gene. All T_1_ progenies of the T_0_ biallelic mutant had biallelic mutations induced by SaCas9 in the *NtFT4* gene (Fig. 1f). Both biallelic mutants and monoallelic mutants were segregated from the monoallelic mutant in the T_1_ generation (Fig. 1f). These results indicate that the mutation induced by SaCas9 is inherited to the next generation.

### Comparing the efficiency of SaCas9 and SpCas9 for genome editing in rice

**Figure 2:**
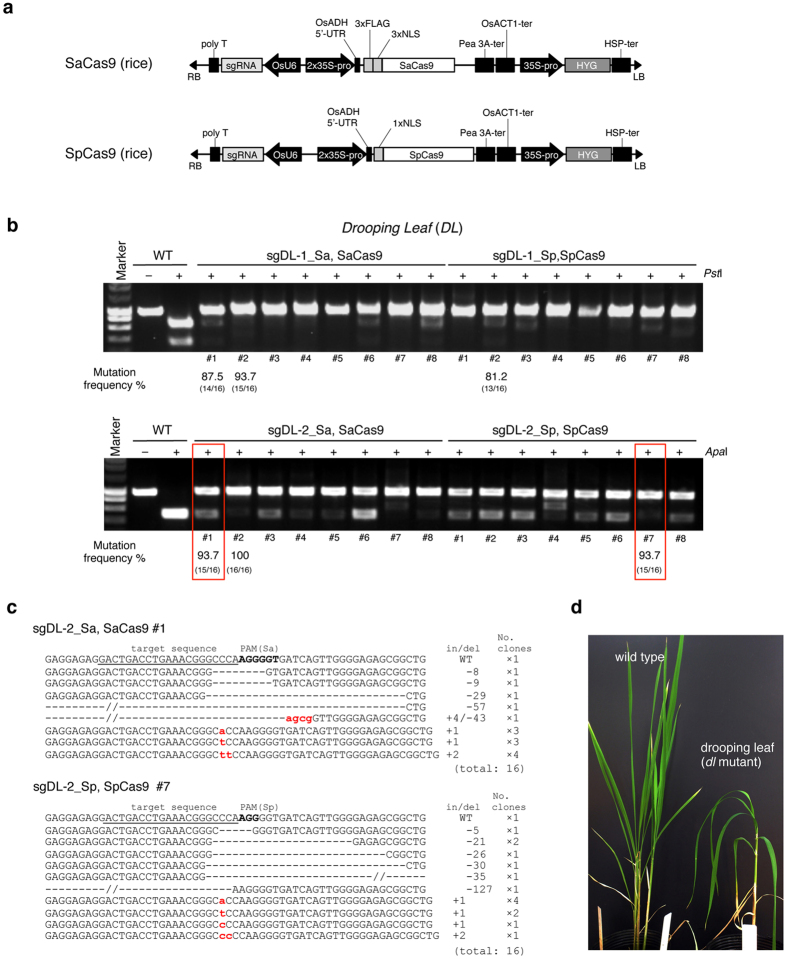
SaCas9 induced mutation in rice. (**a**) Expression construct for *SaCas9* (upper) and *SpCas9* (bottom) in rice. A 3 × FLAG tag and 3 × NLS peptide translationally fused in tandem to the N-terminus of SaCas9. A 1 × FLAG tag was translationally fused to the N-terminus of SpCas9. The OsADH 5′-UTR was added to enhance transcription of the *Cas9* gene. OsADH 5′-UTR: 5′ untranslated region of *Oryza sativa ALCOHOL DEHYDROGENASE* gene. Pea 3A-ter: terminator region of *Pea RBCS-3A* gene, OsACT1-ter: terminator region of *OsACTIN1* gene. (**b**) CAPS analysis of the *OsDL* gene locus in *SaCas9-* and *SpCas9*-expressing calli. Mutation frequency (%) was calculated from the number of clones with mutation versus total number of sequenced clones indicated under the callus number. -: Non-digested PCR products, +: *Pst*I- or *Apa*I-digested PCR products. (**c**) Mutations detected in red rectangle in (**b**). The wild-type sequence is shown at the top with the target sequence underlined and the PAM in bold. DNA deletions and insertions are shown as dashes and lower case red letters. The net change in length and the number of clones are noted to the right of each sequence (+, insertion; −, deletion; ×, number of clones). (**d**) Phenotype of *dl* mutant. *Left* Wild-type plant, *right* a regenerated plant from calli with mutation in *OsDL* gene.

The *SpCas9* gene^24^ optimized for rice codon usage was expressed in rice. It has been reported that an *Arabidopsis thaliana* codon-optimized *SpCas9* gene works as well as the *Oryza sativa* codon-optimized *SpCas9* gene in rice^25^, thus we used *Arabidopsis thaliana* codon-optimized *SaCas9* gene in rice. The expression of both *SaCas9* and *SpCas9* was controlled by a dual CaMV 35S promoter (2 × 35S) (Fig. 2a). The guide RNA was expressed under the control of the *U6-2* promoter derived from *Oryza sativa*^26^. We used the *DROOPING LEAF (DL*) gene for targeted mutagenesis in rice^27^. The *Cas9* gene and gRNA expression vector were both transfected into 1-month-old scutellum-derived rice calli *via* Agrobacterium-mediated transformation. Transformed rice calli were selected on hygromycin plates for 3 weeks (Supplementary Fig. S1b). The mutation frequency was estimated in two ways as follows: (1) by measuring the ratio of calli with mutation to those without, and (2) by measuring the ratio of clones with mutation versus total randomly sequenced clones.

In CAPS analysis, PCR products not digested by restriction enzyme indicated that Cas9 had induced a mutation in the target sequence (Fig. 2b). Mutations were detected in all eight calli expressing *gDL-1_Sa* and *SaCas9* (Fig. 2b, upper left). Also, mutations were detected in all calli expressing *gDL-1_Sp* and *SpCas9* (Fig. 2b, upper right). The same results were obtained in *gDL-2_Sa*- and *SaCas9*-expressing calli and *gDL-2_Sp*- and *SpCas9*-expressing calli (Fig. 2b, lower). We estimated the mutation frequency in independent calli; PCR products derived from independent calli were cloned into plasmids and sequenced. The mutation frequencies of #1 and #2 callus lines expressing *gDL-1_Sa* and *SaCas9* were 87.5% and 93.7%, respectively. The mutation frequency of #2 callus expressing *gDL-1_Sp* and *SpCas9* was 81.2%. The same results were obtained in *gDL-2_Sa, SaCas9*- and *gDL-2_Sp, SpCas9*-expressing callus. These results indicate that SaCas9 could induce mutations in rice just as well as SpCas9, and also that the targeted mutagenesis efficiency of SaCas9 is comparable to that of SpCas9. We examined the patterns of mutation in rice calli caused by SaCas9 and SpCas9 (Fig. 2c). Like SpCas9, small deletions or small insertions close to the PAM sequence were found most often in SaCas9-induced mutations, while large deletions (>25 bp) were rarely detected. We examined the genotype and phenotype of rice plants regenerated from transformed calli that express *gDL-1_Sa* or *gDL-2_Sa* and *SaCas9* (Fig. 2d and Supplementary Fig. S2). The *OsDL* gene is a member of the *YABBY* gene family, and the *dl* mutation causes defects in midrib formation in leaves, resulting in a drooping leaf phenotype^28^; regenerated rice plants possessing a biallelic mutation in the *DL* gene exhibited this phenotype.

### Base preference for ‘T’ at the 6th position of the SaCas9 PAM in plants

**Figure 3:**
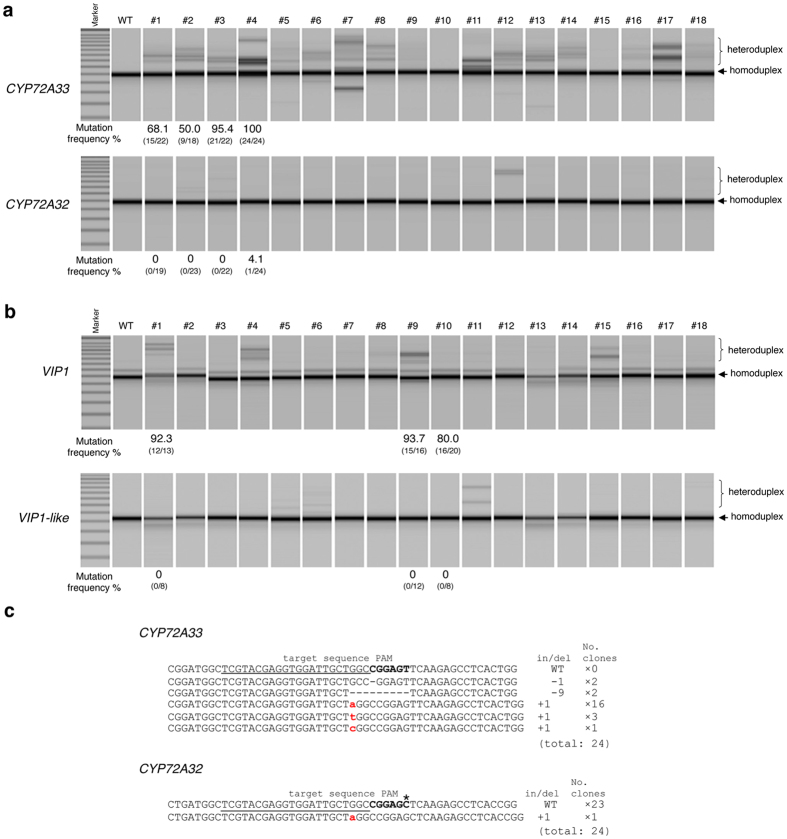
Targeted mutagenesis efficiency in target gene with canonical or non-canonical PAM for SaCas9. (**a**,**b**) Heteroduplex mobility assay of *SaCas9* expression calli. *CYP72A33* and *VIP1* target sequences have canonical PAM (5′-NNGRRT-3′) (upper panel). *CYP72A32* and *VIP1-like* target sequences have non-canonical PAM (5′-NNGRRC-3′ and 5′-NNGRRA-3′) (lower panel). (**c**) Mutations detected in *OsCYP72A33* and *OsCYP72A32* gene loci. The wild-type sequence is shown at the top with the target sequence underlined and the PAM in bold. DNA deletions and insertions are shown as dashes and lower case red letters. The net change in length and the number of clones are noted to the right of each sequence (+, insertion; −, deletion; ×, number of clones). The asterisk (*) indicates the difference from the canonical PAM for SaCas9.

Ran *et al*. reported that the thymine (T) at the 6th position of the SaCas9 PAM 5′-NNGRRT-3′ is not necessary for genome editing in human cells^17^. If this is also the case in plants, the selectivity of potential SaCas9 target sequences can be expanded. To explore this possibility, we selected two pairs of target genes (*CYP72A33* and *OsCYP72A32*, and *OsVIP1* and *OsVIP1-like*). Each pair of genes has a perfect-match target sequence differing only at the 6th position of the PAM sequence (Table 1). We applied a heteroduplex mobility assay (HMA) that has been used previously to detect both transcription activator-like effector nucleases (TALENs)-induced mutation^29^ and CRISPR/Cas9-induced mutation^30^. Genomic DNA was prepared from individual calli expressing *SaCas9* and *gCYP72A33_Sa*. Each of the PCR products from the *CYP72A33* and *CYP72A32* gene loci derived from the same genomic DNA was subjected to HMA. The presence of a heteroduplex structure indicates that the targeted mutation has been introduced into the genomic DNA. Heteroduplex structure was detected in the *CYP72A33* target sequence that has a ‘T’ at the 6th position of PAM (Fig. 3a upper panel). The number of calli exhibiting the presence of the heteroduplex structure was reduced in the *CYP72A32* target sequence, which has a ‘C’ at the 6th position of PAM (Fig. 3a). The frequency of targeted mutagenesis in the *CYP72A33* gene was also reduced to 0–4.1% in the *CYP72A32* gene. The same results were obtained in the *VIP1* gene and *VIP1-like* gene loci (Fig. 3b). The *VIP1* target sequence has a ‘T’ at the 6th position of PAM, while the *VIP1-like* target sequence has an ‘A’ at this position. We examined the patterns of mutation induced in #4 calli at *CYP72A33* and *CYP72A32* gene loci (Fig. 3c). We also analyzed the mutation frequency using other target sequences with non-canonical PAM sequences (Table 4). The results suggested that the ‘T’ at the 6th position of PAM is necessary in order for SaCas9 to recognize and cleave the target sequence in plant cells.

## Discussion

We compared the efficiency of targeted mutagenesis using SaCas9 and SpCas9 on multiple target sequences in rice and tobacco, and showed that SaCas9 works with comparable efficiency to SpCas9. Utilization of SaCas9, which recognizes 5′-NNGRRT-3′ as PAM, expands the selectivity of targeted mutagenesis sites in dicots and monocots and will be a useful addition to the widely used SpCas9. We used target sequences with three (5′-NNGAGT-3′, 5′-NNGGGT-3′, 5′-NNGAAT-3′) out of four PAM patterns for SaCas9 at their 3′ end to induce mutations in tobacco and rice. No obvious difference in targeted mutagenesis efficiency was found between them. These findings suggest that both G and A at the 4th or 5th position of PAM for SaCas9 are equally suitable for genome editing in plants.

The efficiency of targeted mutagenesis with non-canonical PAMs (5′-NNGRRV-3′) is much lower than that with a canonical PAM (5′-NNGRRT-3′) in rice (Fig. 3). Ran *et al*. showed that 5′-NNGRRN-3′ can work as a PAM for SaCas9 in human cells, but that 5′-NNGRRT-3′ was the most efficient in inducing mutation. Kleinstiver *et al*. showed using a bacterial-based negative selection system that three PAMs (5′-NNGAGT-3′, 5′-NNGGGT-3′, 5′-NNGAAT-3′) were more functional than other PAMs^31^. These data are consistent with our experimental data in plant cells. We also recommend that 5′-NNGRRT-3′ be used for SaCas9 PAM in plant cells. Therefore, the sequence 5′-NNGRRT-3′ should be selected as the preferred PAM sequence when designing an efficient targeted mutagenesis strategy in plants using the SaCas9 system.

SpCas9 induces off-target mutations, even at the sites that possess five mismatched bases in the corresponding on-target sequence in human cells^32^,^33^,^34^. In rice cells, SpCas9 with gCDKB2, which is targeted to the *OsCDKB2* gene, induced off-target mutation in the *OsCDKB1* gene, which has two mismatched bases and one of two mismatched bases is located in seed sequence^24^. *NtFT1* gene possesses two mismatched bases at the *NtFT4* target sequence for SaCas9, and one mismatched base in the seed sequence (Fig. 1e). However, we detected no mutation in the *NtFT1* gene (Table 3). The 21- or 22-nt target sequence for SaCas9 is 1 or 2 nt longer than that of SpCas9, and the PAM for SaCas9 is 2 nt longer than that of SpCas9. We have shown that 5′-NNGRRT-3′ is the preferred PAM for targeted mutagenesis using SaCas9 in plants. These results suggest that the probability of off-target mutation induced by SaCas9 might be lower than that induced by SpCas9. This suggestion is supported by data from Friedland *et al*.^35^. Their GUIDE-seq result shows that SaCas9 is more specific than SpCas9 in human cells^35^.

## Methods

### Plasmid construction

The *Arabidopsis thaliana* codon-optimized *SaCas9* gene was synthesized by GeneArt Gene Synthesis (Thermo Fisher Scientific, USA). The backbone of the binary vector used in this study was derived from pRI201-AN (TaKaRa, Japan) and pPZP200 for tobacco and rice, respectively. The Cas9 expression vector in tobacco, pRI201-AN was constructed as follows: (1) the *Arabidopsis thaliana* codon-optimized Cas9 was fused to 3 × FLAG tag (Sigma-Aldrich, USA) and 3 × NLS (nuclear localization signal), respectively. (2) CaMV *35S* promoter (*35S*) and the sequences of *Arabidopsis thaliana* alcohol dehydrogenase (ADH) 5′-untranslated region (UTR) were connected upstream of Cas9, and the terminator sequence of *Arabidopsis thaliana HEAT SHOCK PROTEIN (HSP*) gene was connected downstream of Cas9 (Supplementary Figs S3 and S4). The Cas9 expression vector in rice, pPZP200, was constructed as follows: (1-1) the *Arabidopsis thaliana* codon-optimized SaCas9 was fused to 3 × FLAG tag and 3 × NLS. (1–2) the *Oryza sativa* codon-optimized SpCas9 was fused to 1 × NLS. (2) the dual CaMV 35S promoter (2 × 35S) and the sequences of the rice alcohol dehydrogenase (ADH) 5′-untranslated region (UTR) were connected upstream of Cas9, and the pea RBCS3A (pea3A) terminator sequence and the sequence of the *Oryza sativa ACTIN1 (ACT1*) gene 3′-UTR were connected downstream of Cas9 (Supplementary Figs S5 and S6). The guide RNA expression vector was constructed as follows. (1) The sequence of the *OsU6* promoter and *AtU6*, RNA scaffold and poly (T) were synthesized. (2) The synthesized target sequence (21 nt or 20 nt) was cloned into the *Bbs*I site of the single guide RNA expression vector (Supplementary Fig. S7). The *Pac*I-*Asc*I fragment of the single guide RNA expression vector was subcloned into the Cas9 expression binary vector.

### Transformation of rice callus

Agrobacterium-mediated transformation of rice (*Oryza sativa* L. cv. Nipponbare) using scutellum-derived calli was performed as described previously^36^,^37^. Briefly, one-month-cultured rice calli were infected by Agrobacterium (EHA105 strain). After 3 days of co-cultivation, calli were transferred to callus-induction medium containing 50 mg/L hygromycin B (Wako Pure Chemicals, Osaka, Japan) and 25 mg/L meropenem (Wako Pure Chemicals, Japan). Hygromycin-resistant calli were selected over 4 weeks.

### Transformation of tobacco

Tobacco (*Nicotiana tabacum* L. cv. Petit Havana SR-1) was transformed *via* Agrobacterium-mediated transformation as described previously^38^. Transgenic tobacco plants were regenerated from leaf disks on medium containing 50 mg/L kanamycin (Wako Pure Chemicals, Japan) and 25 mg/L meropenem.

### CAPS analysis

Genomic DNA was extracted from calli, regenerated rice plants or transgenic tobacco shoots using Agencourt Chloro Pure (BECKMAN COULTER, USA), and target loci were amplified by PCR. PCR products were digested by restriction enzymes.

### Heteroduplex mobility assay

Heteroduplex mobility assay (HMA) was applied to detect mutations induced by Cas9 with a microchip electrophoresis system^30^. PCR products were analyzed using MCE-202 MultiNA with a DNA-500 kit (SHIMADZU, Japan).

## Additional Information

Accession codes: NtFT4; AWOKO1087688, DROOPING LEAF; Os03g0215200/LOC_Os03g11600 LOC_Os11g04954, CYP72A32; LOC_Os01g41810, CYP72A33; LOC_Os01g41820, VIP1; LOC_Os12g06520, VIP1-like; LOC_Os11g06170, OsALS; LOC_Os02g30630, OsLIG6; Os01g49180, OsPDS; LOC_Os03g08570, OsPOLQ; LOC_Os12g19370. 

**How to cite this article**: Kaya, H. *et al*. Highly specific targeted mutagenesis in plants using *Staphylococcus aureus* Cas9. *Sci. Rep.* **6**, 26871; doi: 10.1038/srep26871 (2016).

## Supplementary Material

Supplementary Information

## Figures and Tables

**Table 1:** List of target genes, guide RNAs (gRNA), target sequences and PAM sequences used in this study.

target gene	gRNA	target sequence	PAM	reference
*NtFT4*	*gFT4_Sa*	GCCCAAGCAACCCTAACCTGA	GGGAGT	This study
	*gFT4_Sp*	CCCAAGCAACCCTAACCTGA	GGG	This study
*NtPDS*	*gPDS_Sa*	TTGCGATGCCTAACAAGCCAG	GGGART	This study
	*gPDS_Sp*	TGCGATGCCTAACAAGCCAG	GGG	This study
*OsDL1*	*gDL-1_Sa*	GTCTTTTGGGTAGCTGCAGGT	TGGAGT	This study
	*gDL-1_Sp*	TCTTTTGGGTAGCTGCAGGT	TGG	Mikami *et al*.^25^
	*gDL-2_Sa*	GACTGACCTGAAACGGGCCCA	AGGGGT	This study
	*gDL-2_Sp*	ACTGACCTGAAACGGGCCCA	AGG	Mikami *et al*.^25^
*OsCYP72A33*	*gCYP72A33_Sa*	CGTACGAGGTGGATTGCTGGC	CGGAGT	This study
*OsCYP72A32*	*gCYP72A33_Sa*	CGTACGAGGTGGATTGCTGGC	CGGAGC	This study
*OsVIP1*	*gVIP1_Sa*	TAAGAGGAGCGGGTCGATGGA	TGGGGT	This study
*OsVIP1-like*	*gVIP1_Sa*	TAAGAGGAGCGGGTCGATGGA	TGGGGA	This study

Differences from canonical PAM “5-NNGRRT-3” for SaCas9 are underlined.

**Table 2:** Mutation rate of T_0_ regenerated tobacco plants in the target gene.

target gene	Cas9	No. of plants examined	No. of plants with mutations	Mutation rate (%)
*NtPDS*	SaCas9	45	34	75.6
	SpCas9	44	35	79.5
*NtFT4*	SaCas9	43	28	65.1
	SpCas9	22	13	59.1

**Table 3:** Mutation rate of T_0_ regenerated tobacco plants with mismatched bases in the target sequence.

target gene	gRNA	No. of mismatched bases	Cas9	No. of examined plants	No. of plants with mutations	Mutation rate (%)
*NtFT1*	*gNtFT4_Sa*	2	SaCas9	32	0	0
*NtFT2*	*gNtFT4_Sa*	4	SaCas9	32	0	0

**Table 4:** Mutation rate of transformed rice callus in target sequences with non-canonical PAM for SaCas9.

target gene	gRNA	target sequence	PAM	Mutation rate (%)
*OsDL*	*gDL-3_Sa*	GCCAGCTCCTGAATGTTCATG	AGGAAG	0
*OsALS*	*gALS-2_Sa*	GGACCTTGCACTGACTGCAGG	AGGAAC	0
*OsLIG6*	*gLIG6_Sa*	ACGGAGAGCGATTTGGAGTCC	GGGGAG	0
*OsPDS*	*gPDS-1_Sa*	CCGTCCAACCCATTCCTCTGC	AGGAGC	8.3
*OsPOLQ*	*gPOLQ-1_Sa*	TGTATATAGCACAGAGCTCAG	GGGGAA	15.7

Differences from the canonical PAM “5-NNGRRT-3” for SaCas9 are underlined.

**Os*ALS: Oryza sativa acetolactate synthase, OsLIG6: Oryza sativa DNA ligase 6, OsPDS: Oryza sativa phytoene desaturase, OsPOLQ: Oryza sativa DNA polymerase Q.*
