# Supplementary material for: Highly specific targeted mutagenesis in plants using Staphylococcus aureus Cas9
Source: Sci Rep. 2016 May 26;6:26871. doi: 10.1038/srep26871 (PMC4881040; doi:10.1038/srep26871)
Supplement: Supplementary Information [file srep26871-s1.pdf]

Title: Highly specific targeted mutagenesis in plants using *Staphylococcus aureus* Cas9

Author: Hidetaka Kaya[1 †], Masafumi Mikami[1 2 †], Akira Endo[1], Masaki Endo[1], Seiichi Toki[1 2 3 *]

1 Plant Genome Engineering Research Unit, Institute of Agrobiological Sciences, National Agriculture and Food Research Organization, 2-1-2 Kannondai, Tsukuba, Ibaraki 305-8602, Japan

2 Graduate School of Nanobioscience, Yokohama City University, 22-2 Seto, Yokohama, Kanagawa 236 0027, Japan

3 Kihara Institute for Biological Research, Yokohama City University, 641-12 Maioka-cho, Yokohama, Kanagawa 244-0813, Japan

*Corresponding author: Seiichi Toki, Plant Genome Engineering Research Unit, Institute of Agrobiological Sciences, National Agriculture and Food Research Organization, 2-1-2 Kannondai, Tsukuba, Ibaraki 305-8602, Japan, e-mail: stoki@affrc.go.jp, Fax/Tel +81-29-838-8450

[†] These authors contributed equally to this work.

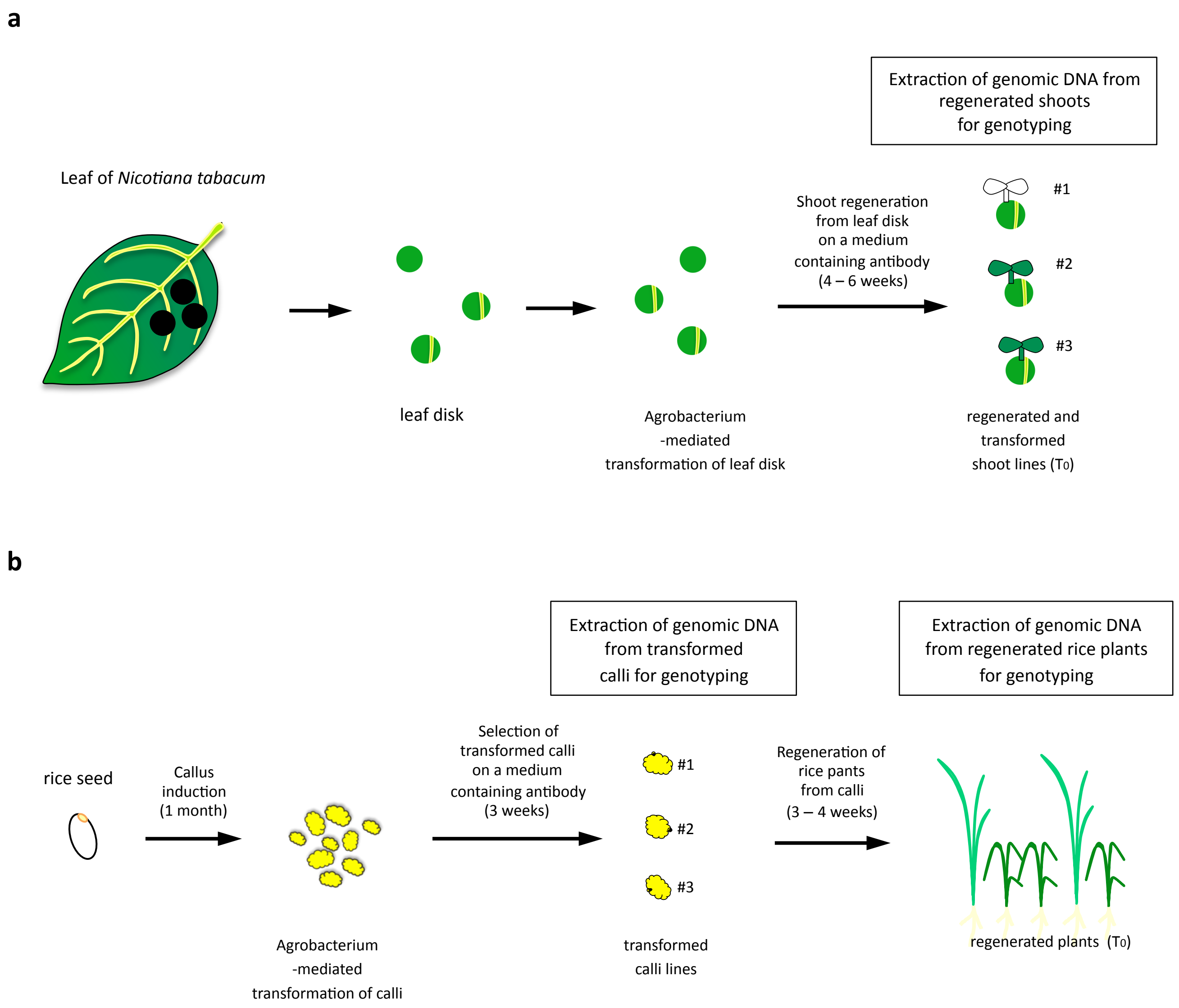

Supplementary Figure 1

Experimental procedure for the preparation of transformed plants.

	sgDL-1_Sa, SaCas9															
callus No.	#2										#4					
Regenerated plants (T_0) No.	1	2	3	4	5	6	7	8	9	10	1	2	3	4	5	6
Genotype	B	M	M	B	B	B	B	B	M	B	B	M	B	M	B	B
Phenotype	D	W	W	D	D	D	D	D	W	D	D	W	D	W	D	D

	sgDL-2_Sa, SaCas9																				
callus No.	#1						#2							#4							
Regenerated plants (T_0) No.	1	2	3	4	5	6	1	2	3	4	5	6	7	1	2	3	4	5	6	7	8
Genotype	B	B	B	B	B	B	B	B	B	B	B	B	B	B	B	B	B	B	B	B	M
Phenotype	D	D	D	D	D	D	D	D	D	D	D	D	D	D	D	D	D	D	D	D	W

Genotype

B: bi-allelic mutation

M: mono-allelic mutation

W: wild type

Phenotype

D: drooping leaf

W: wild type

Supplementary Figure 2

Genotype and phenotype analysis in the rice plants (T_0) regenerated from calli.

Right border

ACTGAAGGCGGGAAACGACAATCTGATCCTGGCGAAAGGGGGATGTGCTGCAAGGCGATTAAGTTGGGTAACGCCAGGGTTTTCCCAGTCACGACGTTGTAAAACGACGGCCAG

*Pac*I *Asc*I 35S-promoter

TGCCAAGCTcttaattaactcgagggcgcgccaagcttagctTGCATGCCTGCAGGTCCCCAGATTAGCCTTTTCAATTTCAGAAAGAATGCTAACCCACAGATGGTTAGAGAG

GCTTACGCAGCAGGTCTCATCAAGACGATCTACCCGAGCAATAATCTCCAGGAAATCAAATACCTTCCCAAGAAGGTTAAAGATGCAGTCAAAAGATTCAGGACTAACTGCATC

AAGAACACAGAGAAAGATATATTTCTCAAGATCAGAAGTACTATTCCAGTATGGACGATTCAAGGCTTGCTTCACAAACCAAGGCAAGTAATAGAGATTGGAGTCTCTAAAAAG

GTAGTTCCCACTGAATCAAAGGCCATGGAGTCAAAGATTCAAATAGAGGACCTAACAGAACTCGCCGTAAAGACTGGCGAACAGTTCATACAGAGTCTCTTACGACTCAATGAC

AAGAAGAAAATCTTCGTCAACATGGTGGAGCACGACACACTTGTCTACTCCAAAAATATCAAAGATACAGTCTCAGAAGACCAAAGGGCAATTGAGACTTTTCAACAAAGGGTA

ATATCCGGAAACCTCCTCGGATTCCATTGCCCAGCTATCTGTCACTTTATTGTGAAGATAGTGGAAAAGGAAGGTGGCTCCTACAAATGCCATCATTGCGATAAAGGAAAGGCC

ATCGTTGAAGATGCCTCTGCCGACAGTGGTCCCAAAGATGGACCCCCACCCACGAGGAGCATCGTGGAAAAAGAAGACGTTCCAACCACGTCTTCAAAGCAAGTGGATTGATGT

GATATCTCCACTGACGTAAGGGATGACGCACAATCCCACTATCCTTCGCAAGACCCTTCCTCTATATAAGGAAGTTCATTTCATTTGGAGAGAACACGGGGGACTCTAGATACA

AtADH5'-UTR 3xFLAG

TCACAATCACACAAAACTAACAAAAGATCAAAAGCAAGTTCTTCACTGTTGATAcatATGGATTACAAGGATCACGATGGTGATTATAAGGATCATGATATTGATTACAAAGAT

3xNLS *A. thaliana* codon-optimized SaCas9

GATGATGCTGATCCTAAGAAGAAGAGGAAAGTTGATCCAAAGAAAAAAAGAAAAGTTGATCCTAAAAAGAAGAGAAAGGTTCACcccgggAAGAGGAACTACATCCTCGGACTC

GATATTGGAATCACCTCTGTGGGATACGGAATCATCGATTACGAGACTAGGGATGTGATCGATGCTGGTGTGAGACTCTTCAAAGAGGCTAACGTTGAGAACAACGAGGGAAGA

AGGTCTAAGAGGGGAGCTAGAAGGCTCAAGAGAAGAAGAAGGCACAGAATCCAGAGGGTGAAGAAGCTCCTCTTCGATTACAACCTCCTCACCGATCACTCTGAGCTTTCTGGA

ATCAACCCTTACGAGGCAAGAGTGAAGGGACTCTCTCAGAAGTTGTCTGAGGAAGAGTTCTCTGCTGCTTTGCTCCACCTTGCTAAGAGAAGGGGTGTGCATAACGTGAACGAG

GTGGAAGAGGATACCGGAAACGAGCTTTCTACCAAAGAGCAGATCAGTAGGAACTCTAAGGCTCTCGAAGAGAAGTACGTGGCAGAGCTTCAGCTTGAGAGACTCAAGAAAGAT

GGTGAGGTGAGGGGATCTATCAACAGGTTCAAGACCTCTGATTACGTGAAAGAAGCTAAGCAGCTCCTCAAGGTGCAGAAGGCTTACCATCAGCTCGATCAGTCTTTCATCGAT

ACCTACATCGATCTCTTGGAGACTAGAAGGACCTACTACGAGGGACCTGGTGAAGGATCTCCATTCGGATGGAAGGATATTAAGGAATGGTACGAGATGCTCATGGGACACTGC

ACTTACTTCCCTGAGGAACTCAGATCAGTGAAGTACGCTTACAACGCTGATCTCTACAACGCTCTTAACGATCTCAACAACCTCGTGATCACCAGGGATGAGAACGAGAAGTTG

GAGTACTATGAGAAGTTCCAGATCATCGAGAACGTGTTCAAGCAAAAGAAGAAGCCTACCCTCAAGCAGATCGCTAAAGAGATCCTTGTTAACGAAGAAGATATTAAGGGATAC

AGGGTGACCTCTACCGGAAAGCCTGAGTTCACTAACCTCAAGGTTTACCACGATATTAAGGATATTACCGCTAGAAAAGAGATTATTGAGAACGCTGAGCTTCTCGATCAAATC

GCTAAGATCCTTACCATCTACCAGTCATCTGAGGATATTCAAGAAGAGTTGACCAACCTCAACTCAGAGCTTACCCAAGAGGAAATCGAGCAAATCTCTAACCTTAAGGGTTAC

ACCGGAACCCACAACCTCTCACTCAAGGCTATCAACCTCATCCTCGATGAGCTTTGGCACACCAACGATAACCAGATCGCAATCTTCAACAGACTCAAGCTCGTGCCTAAGAAA

GTGGATCTCTCTCAGCAGAAAGAGATTCCTACCACCCTCGTGGATGATTTCATCCTCTCACCTGTGGTGAAGAGATCATTCATCCAGTCTATCAAGGTGATCAACGCTATTATC

AAGAAATACGGACTCCCTAACGATATTATCATCGAGTTGGCTAGGGAAAAGAACTCAAAGGATGCTCAAAAGATGATCAACGAGATGCAAAAGAGGAACAGGCAGACCAACGAG

AGGATCGAGGAAATCATCAGGACCACCGGAAAAGAGAACGCTAAGTACCTTATCGAGAAGATCAAGCTCCACGATATGCAAGAGGGAAAGTGCCTCTACTCTCTTGAGGCTATC

CCTCTTGAGGATCTCCTTAACAACCCTTTCAACTACGAAGTGGATCATATCATCCCTAGATCAGTGTCTTTCGATAACTCTTTCAACAACAAGGTGCTCGTGAAGCAAGAAGAA

AACTCTAAGAAGGGAAACAGGACCCCTTTCCAGTACCTCTCTTCATCTGATTCTAAGATCAGTTACGAGACATTCAAGAAACACATCCTCAACCTCGCTAAGGGAAAGGGTAGG

ATCTCAAAGACAAAGAAAGAGTACCTCCTTGAGGAAAGGGATATTAACAGGTTCTCTGTTCAGAAGGATTTCATCAACAGAAACCTTGTGGATACCAGGTACGCTACCAGGGGT

CTTATGAACCTTCTCAGGTCTTACTTCAGGGTGAACAACCTTGATGTGAAGGTGAAGTCAATCAACGGTGGATTCACCTCATTCCTTAGAAGAAAATGGAAGTTTAAGAAAGAG

AGGAACAAGGGTTATAAGCACCACGCTGAGGATGCTCTCATTATCGCTAACGCAGATTTCATTTTCAAAGAGTGGAAGAAACTTGATAAGGCTAAAAAGGTGATGGAAAACCAG

ATGTTCGAGGAAAAGCAGGCTGAGTCTATGCCTGAGATCGAGACTGAGCAAGAGTACAAAGAGATTTTCATCACCCCTCACCAGATCAAGCACATCAAAGATTTCAAGGATTAT

AAGTACTCTCACAGAGTGGATAAGAAGCCAAACAGAGAGCTTATCAACGATACCCTCTACTCAACCAGAAAGGATGATAAGGGTAACACCCTCATCGTGAACAATCTCAACGGA

CTCTACGATAAGGATAACGATAAGCTCAAGAAGCTCATCAACAAGTCTCCAGAGAAGTTGCTCATGTACCACCACGATCCTCAGACCTACCAAAAGCTCAAGTTGATCATGGAA

CAGTACGGTGATGAGAAGAACCCTCTCTACAAGTACTACGAAGAGACTGGAAACTACCTCACCAAGTACTCAAAAAAGGATAATGGACCTGTGATCAAGAAGATTAAGTATTAC

GGAAACAAGCTCAACGCACACCTCGATATTACTGATGATTACCCTAACTCTAGGAACAAAGTGGTGAAGCTCTCACTTAAGCCTTACAGGTTCGATGTGTACCTCGATAACGGT

GTGTACAAGTTCGTGACCGTGAAGAACCTCGATGTTATTAAGAAAGAAAACTATTACGAGGTGAACAGTAAGTGCTACGAGGAAGCTAAGAAGTTGAAGAAGATCAGTAACCAG

GCAGAGTTCATTGCTTCATTCTACAACAACGATCTTATCAAGATTAACGGTGAGCTTTACAGGGTTATCGGAGTGAACAACGATTTGCTCAACAGGATCGAAGTGAACATGATT

GATATTACCTACAGGGAATACCTTGAGAATATGAACGATAAGAGGCCTCCTAGGATCATCAAGACTATCGCTTCTAAGACCCAGAGTATCAAGAAGTACTCTACCGATATTCTC

HSP-terminator

GGAAACCTCTACGAGGTTAAGTCAAAGAAGCACCCTCAGATCATTAAGAAGGGTTGAgagctcTTATGAAGATGAAGATGAAATATTTGGTGTGTCAAATAAAAAGCTAGCTTG

TGTGCTTAAGTTTGTGTTTTTTTCTTGGCTTGTTGTGTTATGAATTTGTGGCTTTTTCTAATATTAAATGAATGTAAGATCTCATTATAATGAATAAACAAATGTTTCTATAAT

*Eco*RI

CCATTGTGAATGTTTTGTTGGATCTCTTCGCATATAACTACTGTATGTGCTATGGTATGGACTATGGAATATGATTAAAGATAAGGAATTGGGGAATTC

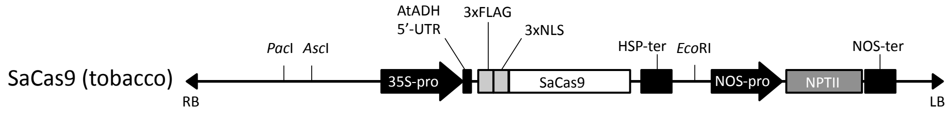

Supplemental Figure 3

SaCas9 expression binary vector for tobacco
backbone vector: pRI-201AN (TaKaRa, Japan)

Right border
ACTGAAGGCGGGAAACGACAATCTGATCCTGGCGAAAGGGGGATGTGCTGCAAGGCGATTAAGTTGGGTAACGCCAGGGTTTTCCCAGTCACGACGTTGTAAAACGACGGCCAG
*Pac*I *Asc*I 35S-promoter
TGCCAAGCTcttaattaactcgagggcgcgccaagcttagctTGCATGCCTGCAGGTCCCCAGATTAGCCTTTTCAATTTCAGAAAGAATGCTAACCCACAGATGGTTAGAGAG
GCTTACGCAGCAGGTCTCATCAAGACGATCTACCCGAGCAATAATCTCCAGGAAATCAAATACCTTCCCAAGAAGGTTAAAGATGCAGTCAAAAGATTCAGGACTAACTGCATC
AAGAACACAGAGAAAGATATATTTCTCAAGATCAGAAGTACTATTCCAGTATGGACGATTCAAGGCTTGCTTCACAAACCAAGGCAAGTAATAGAGATTGGAGTCTCTAAAAAG
GTAGTTCCCACTGAATCAAAGGCCATGGAGTCAAAGATTCAAATAGAGGACCTAACAGAACTCGCCGTAAAGACTGGCGAACAGTTCATACAGAGTCTCTTACGACTCAATGAC
AAGAAGAAAATCTTCGTCAACATGGTGGAGCACGACACACTTGTCTACTCCAAAAATATCAAAGATACAGTCTCAGAAGACCAAAGGGCAATTGAGACTTTTCAACAAAGGGTA
ATATCCGGAAACCTCCTCGGATTCCATTGCCCAGCTATCTGTCACTTTATTGTGAAGATAGTGGAAAAGGAAGGTGGCTCCTACAAATGCCATCATTGCGATAAAGGAAAGGCC
ATCGTTGAAGATGCCTCTGCCGACAGTGGTCCCAAAGATGGACCCCCACCCACGAGGAGCATCGTGGAAAAAGAAGACGTTCCAACCACGTCTTCAAAGCAAGTGGATTGATGT
GATATCTCCACTGACGTAAGGGATGACGCACAATCCCACTATCCTTCGCAAGACCCTTCCTCTATATAAGGAAGTTCATTTCATTTGGAGAGAACACGGGGGACTCTAGATACA
AtADH5'-UTR 3xFLAG
TCACAATCACACAAAACTAACAAAAGATCAAAAGCAAGTTCTTCACTGTTGATAcatATGGATTACAAGGATCACGATGGTGATTATAAGGATCATGATATTGATTACAAAGAT
3xNLS *A. thaliana* codon-optimized *SpCas9*
GATGATGCTGATCCTAAGAAGAAGAGGAAAGTTGATCCAAAGAAAAAAAGAAAAGTTGATCCTAAAAAGAAGAGAAAGGTTCACcccgggGATAAGAAGTACTCTATCGGACTC
GATATCGGAACTAACTCTGTGGGATGGGCTGTGATCACCGATGAGTACAAGGTGCCATCTAAGAAGTTCAAGGTTCTCGGAAACACCGATAGGCACTCTATCAAGAAAAACCTT
ATCGGTGCTCTCCTCTTCGATTCTGGTGAAACTGCTGAGGCTACCAGACTCAAGAGAACCGCTAGAAGAAGGTACACCAGAAGAAAGAACAGGATCTGCTACCTCCAAGAGATC
TTCTCTAACGAGATGGCTAAAGTGGATGATTCATTCTTCCACAGGCTCGAAGAGTCATTCCTCGTGGAAGAAGATAAGAAGCACGAGAGGCACCCTATCTTCGGAAACATCGTT
GATGAGGTGGCATACCACGAGAAGTACCCTACTATCTACCACCTCAGAAAGAAGCTCGTTGATTCTACTGATAAGGCTGATCTCAGGCTCATCTACCTCGCTCTCGCTCACATG
ATCAAGTTCAGAGGACACTTCCTCATCGAGGGTGATCTCAACCCTGATAACTCTGATGTGGATAAGTTGTTCATCCAGCTCGTGCAGACCTACAACCAGCTTTTCGAAGAGAAC
CCTATCAACGCTTCAGGTGTGGATGCTAAGGCTATCCTCTCTGCTAGGCTCTCTAAGTCAAGAAGGCTTGAGAACCTCATTGCTCAGCTCCCTGGTGAGAAGAAGAACGGACTT
TTCGGAAACTTGATCGCTCTCTCTCTCGGACTCACCCCTAACTTCAAGTCTAACTTCGATCTCGCTGAGGATGCAAAGCTCCAGCTCTCAAAGGATACCTACGATGATGATCTC
GATAACCTCCTCGCTCAGATCGGAGATCAGTACGCTGATTTGTTCCTCGCTGCTAAGAACCTCTCTGATGCTATCCTCCTCAGTGATATCCTCAGAGTGAACACCGAGATCACC
AAGGCTCCACTCTCAGCTTCTATGATCAAGAGATACGATGAGCACCACCAGGATCTCACACTTCTCAAGGCTCTTGTTAGACAGCAGCTCCCAGAGAAGTACAAAGAGATTTTC
TTCGATCAGTCTAAGAACGGATACGCTGGTTACATCGATGGTGGTGCATCTCAAGAAGAGTTCTACAAGTTCATCAAGCCTATCCTCGAGAAGATGGATGGAACCGAGGAACTC
CTCGTGAAGCTCAATAGAGAGGATCTTCTCAGAAAGCAGAGGACCTTCGATAACGGATCTATCCCTCATCAGATCCACCTCGGAGAGTTGCACGCTATCCTTAGAAGGCAAGAG
GATTTCTACCCATTCCTCAAGGATAACAGGGAAAAGATTGAGAAGATTCTCACCTTCAGAATCCCTTACTACGTGGGACCTCTCGCTAGAGGAAACTCAAGATTCGCTTGGATG
ACCAGAAAGTCTGAGGAAACCATCACCCCTTGGAACTTCGAAGAGGTGGTGGATAAGGGTGCTAGTGCTCAGTCTTTCATCGAGAGGATGACCAACTTCGATAAGAACCTTCCA
AACGAGAAGGTGCTCCCTAAGCACTCTTTGCTCTACGAGTACTTCACCGTGTACAACGAGTTGACCAAGGTTAAGTACGTGACCGAGGGAATGAGGAAGCCTGCTTTTTTGTCA
GGTGAGCAAAAGAAGGCTATCGTTGATCTCTTGTTCAAGACCAACAGAAAGGTGACCGTGAAGCAGCTCAAAGAGGATTACTTCAAGAAAATCGAGTGCTTCGATTCAGTTGAG
ATTTCTGGTGTTGAGGATAGGTTCAACGCATCTCTCGGAACCTACCACGATCTCCTCAAGATCATTAAGGATAAGGATTTCTTGGATAACGAGGAAAACGAGGATATCTTGGAG
GATATCGTTCTTACCCTCACCCTCTTTGAAGATAGAGAGATGATTGAAGAAAGGCTCAAGACCTACGCTCATCTCTTCGATGATAAGGTGATGAAGCAGTTGAAGAGAAGAAGA
TACACTGGTTGGGGAAGGCTCTCAAGAAAGCTCATTAACGGAATCAGGGATAAGCAGTCTGGAAAGACAATCCTTGATTTCCTCAAGTCTGATGGATTCGCTAACAGAAACTTC
ATGCAGCTCATCCACGATGATTCTCTCACCTTTAAAGAGGATATCCAGAAGGCTCAGGTTTCAGGACAGGGTGATAGTCTCCATGAGCATATCGCTAACCTCGCTGGATCTCCT
GCAATCAAGAAGGGAATCCTCCAGACTGTGAAGGTTGTGGATGAGTTGGTGAAGGTGATGGGAAGGCATAAGCCTGAGAACATCGTGATCGAAATGGCTAGAGAGAACCAGACC
ACTCAGAAGGGACAGAAGAACTCTAGGGAAAGGATGAAGAGGATCGAGGAAGGTATCAAAGAGCTTGGATCTCAGATCCTCAAAGAGCACCCTGTTGAGAACACTCAGCTCCAG
AATGAGAAGCTCTACCTCTACTACCTCCAGAACGGAAGGGATATGTATGTGGATCAAGAGTTGGATATCAACAGGCTCTCTGATTACGATGTTGATCATATCGTGCCACAGTCA
TTCTTGAAGGATGATTCTATCGATAACAAGGTGCTCACCAGGTCTGATAAGAACAGGGGTAAGAGTGATAACGTGCCAAGTGAAGAGGTTGTGAAGAAAATGAAGAACTATTGG
AGGCAGCTCCTCAACGCTAAGCTCATCACTCAGAGAAAGTTCGATAACTTGACTAAGGCTGAGAGGGGAGGACTCTCTGAATTGGATAAGGCAGGATTCATCAAGAGGCAGCTT
GTGGAAACCAGGCAGATCACTAAGCACGTTGCACAGATCCTCGATTCTAGGATGAACACCAAGTACGATGAGAACGATAAGTTGATCAGGGAAGTGAAGGTTATCACCCTCAAG
TCAAAGCTCGTGTCTGATTTCAGAAAGGATTTCCAATTCTACAAGGTGAGGGAAATCAACAACTACCACCACGCTCACGATGCTTACCTTAACGCTGTTGTTGGAACCGCTCTC
ATCAAGAAGTATCCTAAGCTCGAGTCAGAGTTCGTGTACGGTGATTACAAGGTGTACGATGTGAGGAAGATGATCGCTAAGTCTGAGCAAGAGATCGGAAAGGCTACCGCTAAG
TATTTCTTCTACTCTAACATCATGAATTTCTTCAAGACCGAGATTACCCTCGCTAACGGTGAGATCAGAAAGAGGCCACTCATCGAGACAAACGGTGAAACAGGTGAGATCGTG
TGGGATAAGGGAAGGGATTTCGCTACCGTTAGAAAGGTGCTCTCTATGCCACAGGTGAACATCGTTAAGAAAACCGAGGTGCAGACCGGTGGATTCTCTAAAGAGTCTATCCTC
CCTAAGAGGAACTCTGATAAGCTCATTGCTAGGAAGAAGGATTGGGACCCTAAGAAATACGGTGGTTTCGATTCTCCTACCGTGGCTTACTCTGTTCTCGTTGTGGCTAAGGTT
GAGAAGGGAAAGAGTAAGAAGCTCAAGTCTGTTAAGGAACTTCTCGGAATCACTATCATGGAAAGGTCATCTTTCGAGAAGAACCCAATCGATTTCCTCGAGGCTAAGGGATAC
AAAGAGGTTAAGAAGGATCTCATCATCAAGCTCCCAAAGTACTCACTCTTCGAACTCGAGAACGGTAGAAAGAGGATGCTCGCTTCTGCTGGTGAGCTTCAAAAGGGAAACGAG
CTTGCTCTCCCATCTAAGTACGTTAACTTTCTTTACCTCGCTTCTCACTACGAGAAGTTGAAGGGATCTCCAGAAGATAACGAGCAGAAGCAACTTTTCGTTGAGCAGCACAAG
CACTACTTGGATGAGATCATCGAGCAGATCTCTGAGTTCTCTAAAAGGGTGATCCTCGCTGATGCAAACCTCGATAAGGTGTTGTCTGCTTACAACAAGCACAGAGATAAGCCT
ATCAGGGAACAGGCAGAGAACATCATCCATCTCTTCACCCTTACCAACCTCGGTGCTCCTGCTGCTTTCAAGTACTTCGATACAACCATCGATAGGAAGAGATACACCTCTACC
HSP-terminator
AAAGAAGTGCTCGATGCTACCCTCATCCATCAGTCTATCACTGGACTCTACGAGACTAGGATCGATCTCTCACAGCTCGGTGGTGATTGAgagctcTTATGAAGATGAAGATGA
AATATTTGGTGTGTCAAATAAAAAGCTAGCTTGTGTGCTTAAGTTTGTGTTTTTTTCTTGGCTTGTTGTGTTATGAATTTGTGGCTTTTTCTAATATTAAATGAATGTAAGATC
TCATTATAATGAATAAACAAATGTTTCTATAATCCATTGTGAATGTTTTGTTGGATCTCTTCGCATATAACTACTGTATGTGCTATGGTATGGACTATGGAATATGATTAAAGA
*Eco*RI
TAAGGAATTGGGGAATTC

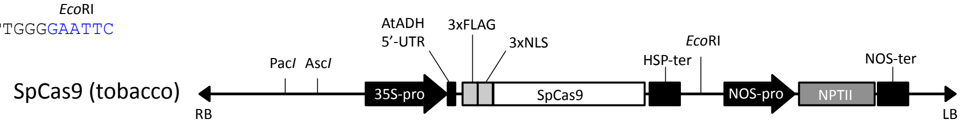

Supplemental Figure 4

SpCas9 expression binary vector for tobacco

backbone vector: pRI-201AN (TaKaRa, Japan)

Right border

TAAACGCTCTTTTCTCTTAGGTTTACccgccaatatatcctgtcaaacactgatagtttaaactgaaggcgggaaacgacaatctgatccaagctcaagctccaatacgcaaa

ccgcctctccccgcgcgttggccgattcattaatgcagctggcacgacaggtttcccgactggaaagcgggcagtgagcgcaacgcaattaatgtgagttagctcactcatta

ggcaccccaggctttacactttatgcttccggctcgtatgttgtgtggaattgtgagcggataacaatttcacacaggaaacagctatgaccatgattacGAATTTCAGGTGA

CTGATAGTGACCTGTTCGTTGCAACAAATTGATGAGCAATGCTTTTTTATAATGCCAACTTTGTACAAAAAAGCAGGCggcgcgtCCTGCAGGATAGGGATAACAGGGTAATg

*Asc*I

gcgcgccAACTCAATGCAAAACGAAAAAAAAAAGCACCACCGACTCGGTGCCACTTTTTCAAGTTGATAACGGACTAGCCTTATTTTAACTTGCTATTTCTAGCTCTAAAACc

gagacctcggtctccTGCCACGGATCATCTGCACAACTCTTTTAAAtCAGCTTTGATCTATGTGGATAGCCGAGGTGGTACTAATACTAGTCTTTGTTGTCGTCCAATTGCGT

AATGGGCCGGCCCATACTGCAATACATGTCCTGAAAGGCTTCATGGCCCACTACGAAATGCTTTTCTCCTACAGTTTATCTTACTtCttCACATCACGTGGTTTCCAACGTAC

CCAGTGTTCCCGGCTTCCAGCATTTGCTGGTAGCACCAGTAGAAGACGCCTGTCtTGTGCTATGGTCCCTGACTGCACATCTGATTCCTCCAAGATCCATGCATGCCTGATAA

*Pac*I

CTTTAAGTTGCTTCAGAAGAACTTTAAGTGATCTGTTCGTATGTTTAAAGATtCCTTGAttaattaaATTACCCTGTTATCCCTATCCTGCAGGattaaCACCCAACTTTTCT

2x35S promoter

ATCCaagcttgccaacatggtggagcacgacactctcgtctactccaagaatatcaaagatacagtctcagaagaccaaagggctattgagacttttcaacaaagggtaatat

cgggaaacctcctcggattccattgcccagctatctgtcacttcatcaaaaggacagtagaaaaggaaggtggcacctacaaatgccatcattgcgataaaggaaaggctatc

gttcaagatgcctctgccgacagtggtcccaaagatggacccccacccacgaggagcatcgtggaaaaagaagacgttccaaccacgtcttcaaagcaagtggattgatgtga

taacatggtggagcacgacactctcgtctactccaagaatatcaaagatacagtctcagaagaccaaagggctattgagacttttcaacaaagggtaatatcgggaaacctcc

tcggattccattgcccagctatctgtcacttcatcaaaaggacagtagaaaaggaaggtggcacctacaaatgccatcattgcgataaaggaaaggctatcgttcaagatgcc

tctgccgacagtggtcccaaagatggacccccacccacgaggagcatcgtggaaaaagaagacgttccaaccacgtcttcaaagcaagtggattgatgtgatatctccactga

OsADH5'-UTR

cgtaagggatgacgcacaatcccactatccttcgcaagacccttcctctatataaggaagttcatttcatttggagaggccggtctagaGAATTCCAAGCAACGAACTGCGAG

3xFLAG

TGATTCAAGAAAAAAGAAAACCTGAGCTTTCGATCTCTACGGAGTGGTTTCTTGTTCTTTGAAAAAGAGGGGGATTAcatATGGATTACAAGGATCACGATGGTGATTATAAG

3xNLS

GATCATGATATTGATTACAAAGATGATGATGCTGATCCTAAGAAGAAGAGGAAAGTTGATCCAAAGAAAAAAAGAAAAGTTGATCCTAAAAAGAAGAGAAAGGTTCACcccgg

A. thaliana codon-optimized *SaCas9*

gAAGAGGAACTACATCCTCGGACTCGATATTGGAATCACCTCTGTGGGATACGGAATCATCGATTACGAGACTAGGGATGTGATCGATGCTGGTGTGAGACTCTTCAAAGAGG

CTAACGTTGAGAACAACGAGGGAAGAAGGTCTAAGAGGGGAGCTAGAAGGCTCAAGAGAAGAAGAAGGCACAGAATCCAGAGGGTGAAGAAGCTCCTCTTCGATTACAACCTC

CTCACCGATCACTCTGAGCTTTCTGGAATCAACCCTTACGAGGCAAGAGTGAAGGGACTCTCTCAGAAGTTGTCTGAGGAAGAGTTCTCTGCTGCTTTGCTCCACCTTGCTAA

GAGAAGGGGTGTGCATAACGTGAACGAGGTGGAAGAGGATACCGGAAACGAGCTTTCTACCAAAGAGCAGATCAGTAGGAACTCTAAGGCTCTCGAAGAGAAGTACGTGGCAG

AGCTTCAGCTTGAGAGACTCAAGAAAGATGGTGAGGTGAGGGGATCTATCAACAGGTTCAAGACCTCTGATTACGTGAAAGAAGCTAAGCAGCTCCTCAAGGTGCAGAAGGCT

TACCATCAGCTCGATCAGTCTTTCATCGATACCTACATCGATCTCTTGGAGACTAGAAGGACCTACTACGAGGGACCTGGTGAAGGATCTCCATTCGGATGGAAGGATATTAA

GGAATGGTACGAGATGCTCATGGGACACTGCACTTACTTCCCTGAGGAACTCAGATCAGTGAAGTACGCTTACAACGCTGATCTCTACAACGCTCTTAACGATCTCAACAACC

TCGTGATCACCAGGGATGAGAACGAGAAGTTGGAGTACTATGAGAAGTTCCAGATCATCGAGAACGTGTTCAAGCAAAAGAAGAAGCCTACCCTCAAGCAGATCGCTAAAGAG

ATCCTTGTTAACGAAGAAGATATTAAGGGATACAGGGTGACCTCTACCGGAAAGCCTGAGTTCACTAACCTCAAGGTTTACCACGATATTAAGGATATTACCGCTAGAAAAGA

GATTATTGAGAACGCTGAGCTTCTCGATCAAATCGCTAAGATCCTTACCATCTACCAGTCATCTGAGGATATTCAAGAAGAGTTGACCAACCTCAACTCAGAGCTTACCCAAG

AGGAAATCGAGCAAATCTCTAACCTTAAGGGTTACACCGGAACCCACAACCTCTCACTCAAGGCTATCAACCTCATCCTCGATGAGCTTTGGCACACCAACGATAACCAGATC

GCAATCTTCAACAGACTCAAGCTCGTGCCTAAGAAAGTGGATCTCTCTCAGCAGAAAGAGATTCCTACCACCCTCGTGGATGATTTCATCCTCTCACCTGTGGTGAAGAGATC

ATTCATCCAGTCTATCAAGGTGATCAACGCTATTATCAAGAAATACGGACTCCCTAACGATATTATCATCGAGTTGGCTAGGGAAAAGAACTCAAAGGATGCTCAAAAGATGA

TCAACGAGATGCAAAAGAGGAACAGGCAGACCAACGAGAGGATCGAGGAAATCATCAGGACCACCGGAAAAGAGAACGCTAAGTACCTTATCGAGAAGATCAAGCTCCACGAT

ATGCAAGAGGGAAAGTGCCTCTACTCTCTTGAGGCTATCCCTCTTGAGGATCTCCTTAACAACCCTTTCAACTACGAAGTGGATCATATCATCCCTAGATCAGTGTCTTTCGA

TAACTCTTTCAACAACAAGGTGCTCGTGAAGCAAGAAGAAAACTCTAAGAAGGGAAACAGGACCCCTTTCCAGTACCTCTCTTCATCTGATTCTAAGATCAGTTACGAGACAT

TCAAGAAACACATCCTCAACCTCGCTAAGGGAAAGGGTAGGATCTCAAAGACAAAGAAAGAGTACCTCCTTGAGGAAAGGGATATTAACAGGTTCTCTGTTCAGAAGGATTTC

ATCAACAGAAACCTTGTGGATACCAGGTACGCTACCAGGGGTCTTATGAACCTTCTCAGGTCTTACTTCAGGGTGAACAACCTTGATGTGAAGGTGAAGTCAATCAACGGTGG

ATTCACCTCATTCCTTAGAAGAAAATGGAAGTTTAAGAAAGAGAGGAACAAGGGTTATAAGCACCACGCTGAGGATGCTCTCATTATCGCTAACGCAGATTTCATTTTCAAAG

AGTGGAAGAAACTTGATAAGGCTAAAAAGGTGATGGAAAACCAGATGTTCGAGGAAAAGCAGGCTGAGTCTATGCCTGAGATCGAGACTGAGCAAGAGTACAAAGAGATTTTC

ATCACCCCTCACCAGATCAAGCACATCAAAGATTTCAAGGATTATAAGTACTCTCACAGAGTGGATAAGAAGCCAAACAGAGAGCTTATCAACGATACCCTCTACTCAACCAG

AAAGGATGATAAGGGTAACACCCTCATCGTGAACAATCTCAACGGACTCTACGATAAGGATAACGATAAGCTCAAGAAGCTCATCAACAAGTCTCCAGAGAAGTTGCTCATGT

ACCACCACGATCCTCAGACCTACCAAAAGCTCAAGTTGATCATGGAACAGTACGGTGATGAGAAGAACCCTCTCTACAAGTACTACGAAGAGACTGGAAACTACCTCACCAAG

TACTCAAAAAAGGATAATGGACCTGTGATCAAGAAGATTAAGTATTACGGAAACAAGCTCAACGCACACCTCGATATTACTGATGATTACCCTAACTCTAGGAACAAAGTGGT

GAAGCTCTCACTTAAGCCTTACAGGTTCGATGTGTACCTCGATAACGGTGTGTACAAGTTCGTGACCGTGAAGAACCTCGATGTTATTAAGAAAGAAAACTATTACGAGGTGA

ACAGTAAGTGCTACGAGGAAGCTAAGAAGTTGAAGAAGATCAGTAACCAGGCAGAGTTCATTGCTTCATTCTACAACAACGATCTTATCAAGATTAACGGTGAGCTTTACAGG

GTTATCGGAGTGAACAACGATTTGCTCAACAGGATCGAAGTGAACATGATTGATATTACCTACAGGGAATACCTTGAGAATATGAACGATAAGAGGCCTCCTAGGATCATCAA

GACTATCGCTTCTAAGACCCAGAGTATCAAGAAGTACTCTACCGATATTCTCGGAAACCTCTACGAGGTTAAGTCAAAGAAGCACCCTCAGATCATTAAGAAGGGTTGAgagc

Pea 3A-ter

tccgatccaggcctcccagctttcgtccgtatcatcggtttcgacaacgttcgtcaagttcaatgcatcagtttcattgcccacacaccagaatcctactaagtttgagtatt

atggcattggaaaagctgttttcttctatcatttgttctgcttgtaatttactgtgttctttcagtttttgttttcggacatcaaaatgcaaatggatggataagagttaata

aatgatatggtccttttgttcattctcaaattattattatctgttgtttttactttaatgggttgaatttaagtaagaaaggaactaacagtgtgatattaaggtgcaatgtt

agacatataaaacagtctttcacctctctttggttatgtcttgaattggtttgtttcttcacttatctgtgtaatcaagtttactatgagtctatgatcaagtaattatgcaa

OsACT1-ter

tcaagttaagtacagtataggctttggtaccttaGCCCCTAGGtaattcttcggacccaagaatgctaagccaagaggagctgttatcgccgtcctcctgcttgtttctctct

ttttgttgctgtttcttcattagcgtggacaaagttttcaaccggcctatctgttatcattttcttctattcaaagactgtaatacctattgctacctg
tggttctcacttgtgattttggacacatatgttcggtttattcaaatttaatcagatgcctgatgagggtaccagaaaaaatacgtgttctggttgttt
ttgagttgcgattattctatgaaatgaataacatcgaagttatcatcccagtattttcgcatgaatgttcttttcttctgtcttgtgcatcagtgatct
agtgcatgggagtttgtattgtgatgttcgacatcacgtaacttccactttgcctttgctgttcgatattttaatgacatgtcacacacacttctgata
cttttctttcttggctattgtgccagcatgatgcaagatgcatcacagcatcagatatattctcatcgtcaggctttagcagcacacgagcacgctttg
ccgcttaaaagttgtacggcgcagcttagacatcccctgtagaagtgataatcttttcacttttccttaaacaaattgagaggggaaatggaaccatgt
ggatcagagaagcttttgtttctttacacaagaatatttggtacagtgggggtcctatgttcgtgggttcgtggcttggctgcctgtcttcaaccaagt
gttttcagttcaacatgttagcgtgtagaaagagcacaattctgtttatctccaaggtaaaatgtggcattctgttaaagaacatgatcctgccaattt
tttaagtttcaatggaagaggaatgtaaagctttctatggtttgtgtacacaacacagtggaagaggagtgcaagctttctatggtttgtgtgcgcgtt
gtgtgtcagcacttcaattttgttagaaaatgaaagaaaaaaaaggatgatcatgcttatagtaaatcactctttttcctcgccttctgtacgttttga
cttgacaagattttaaaatctgtacatgacctttgttttaaaattactttatgtatttccatctttcaagttatgcagatgtcatcacaaattgttaca
ccaatcaccaggctggctgtttatatattatcagaccaggctatatagagtatactatactaactgttcatattatctggaaatcttgcttgctacttg
agcggtaaaagggtatagatatgagggtcccc

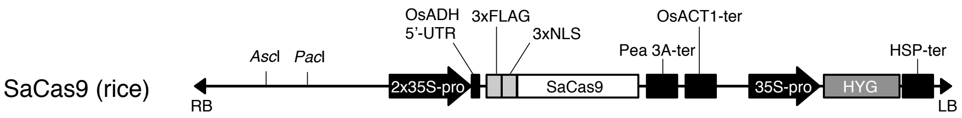

Supplemental Figure 5

SaCas9 expression binary vector for rice

backbone vector: pPZP200

Right border
TAAACGCTCTTTTCTCTTAGGTTTACccgccaatatatcctgtcaaacactgatagtttaaactgaaggcgggaaacgacaatctgatccaagctcaagctccaatacgcaaac
cgcctctccccgcgcgttggccgattcattaatgcagctggcacgacaggtttcccgactggaaagcgggcagtgagcgcaacgcaattaatgtgagttagctcactcattagg
caccccaggctttacactttatgcttccggctcgtatgttgtgtggaattgtgagcggataacaatttcacacaggaaacagctatgaccatgattacGAATTTCAGGTGACTG
*Asc*I
ATAGTGACCTGTTCGTTGCAACAAATTGATGAGCAATGCTTTTTTATAATGCCAACTTTGTACAAAAAAGCAGGCggcgcgtCCTGCAGGATAGGGATAACAGGGTAATggcgc
gccAACTCAATGCAAAACGAAAAAAAAAAGCACCACCGACTCGGTGCCACTTTTTCAAGTTGATAACGGACTAGCCTTATTTTAACTTGCTATTTCTAGCTCTAAAACcgagac
ctcggtctccTGCCACGGATCATCTGCACAACTCTTTTAAAtCAGCTTTGATCTATGTGGATAGCCGAGGTGGTACTAATACTAGTCTTTGTTGTCGTCCAATTGCGTAATGGG
CCGGCCCATACTGCAATACATGTCCTGAAAGGCTTCATGGCCCACTACGAAATGCTTTTCTCCTACAGTTTATCTTACTtCttCACATCACGTGGTTTCCAACGTACCCAGTGT
TCCCGGCTTCCAGCATTTGCTGGTAGCACCAGTAGAAGACGCCTGTCtTGTGCTATGGTCCCTGACTGCACATCTGATTCCTCCAAGATCCATGCATGCCTGATAACTTTAAGT
*Pac*I
TGCTTCAGAAGAACTTTAAGTGATCTGTTCGTATGTTTAAAGATtCCTTGAttaattaaATTACCCTGTTATCCCTATCCTGCAGGattaaCACCCAACTTTTCTATCCaagct
2x35S promoter
tgccaacatggtggagcacgacactctcgtctactccaagaatatcaaagatacagtctcagaagaccaaagggctattgagacttttcaacaaagggtaatatcgggaaacct
cctcggattccattgcccagctatctgtcacttcatcaaaaggacagtagaaaaggaaggtggcacctacaaatgccatcattgcgataaaggaaaggctatcgttcaagatgc
ctctgccgacagtggtcccaaagatggacccccacccacgaggagcatcgtggaaaaagaagacgttccaaccacgtcttcaaagcaagtggattgatgtgataacatggtgga
gcacgacactctcgtctactccaagaatatcaaagatacagtctcagaagaccaaagggctattgagacttttcaacaaagggtaatatcgggaaacctcctcggattccattg
cccagctatctgtcacttcatcaaaaggacagtagaaaaggaaggtggcacctacaaatgccatcattgcgataaaggaaaggctatcgttcaagatgcctctgccgacagtgg
tcccaaagatggacccccacccacgaggagcatcgtggaaaaagaagacgttccaaccacgtcttcaaagcaagtggattgatgtgatatctccactgacgtaagggatgacgc
OsADH5′-UTR
acaatcccactatccttcgcaagacccttcctctatataaggaagttcatttcatttggagaggccggtctagaGAATTCCAAGCAACGAACTGCGAGTGATTCAAGAAAAAAG
1xNLS
AAAACCTGAGCTTTCGATCTCTACGGAGTGGTTTCTTGTTCTTTGAAAAAGAGGGGGATTACATATGGCTCCTAAGAAGAAGCGGAAGGTTGGTATTCACGGGGTGCCTGCGGC
Oryza sativa codon-optimized *SpCas9*
TATGGACAAGAAGTACTCGATCGGGCTGGACATCGGAACAAATTCTGTAGGCTGGGCTGTAATAACCGATGAGTACAAGGTGCCCTCTAAAAAATTTAAGGTCCTTGGCAATAC
GGATAGACATTCCATAAAGAAGAATCTTATCGGTGCGCTGCTCTTTGACAGCGGCGAGACCGCGGAGGCGACCCGGTTGAAACGCACCGCGAGACGCCGTTACACAAGGCGTAA
GAATAGAATCTGTTATCTCCAGGAGATATTCTCTAATGAAATGGCGAAGGTAGACGATTCCTTCTTTCACCGTCTGGAGGAAAGTTTTCTCGTTGAGGAAGATAAGAAACATGA
AAGACACCCGATCTTCGGAAACATTGTCGACGAGGTCGCTTATCATGAAAAGTACCCTACCATCTACCATCTTAGAAAGAAACTTGTTGACAGCACGGATAAGGCTGATCTCAG
GCTGATATACCTGGCTCTGGCACATATGATTAAGTTCAGAGGGCATTTCCTTATCGAAGGCGACCTGAATCCAGATAATTCAGATGTAGACAAGCTCTTCATTCAACTTGTGCA
GACTTATAATCAGCTCTTCGAAGAAAATCCAATAAACGCGTCGGGTGTAGACGCAAAGGCCATACTGTCCGCTAGGCTTTCTAAGTCACGTAGACTTGAGAATCTCATTGCCCA
ACTCCCCGGCGAGAAGAAGAACGGCTTGTTTGGAAATCTGATAGCGCTGTCCCTGGGTCTTACACCAAATTTCAAGAGTAATTTCGATTTGGCAGAAGATGCTAAGTTGCAGCT
CAGTAAAGACACCTACGATGACGATCTTGATAATTTGTTGGCTCAGATTGGCGATCAGTATGCAGATCTTTTCTTGGCCGCTAAGAATTTGTCTGATGCAATTCTGCTTAGCGA
CATTTTGAGGGTTAATACAGAAATCACCAAGGCACCCTTGTCGGCGTCAATGATAAAGAGGTATGATGAGCACCACCAAGACCTGACGCTCCTCAAGGCTCTTGTTCGGCAGCA
ATTGCCGGAGAAGTACAAAGAGATCTTCTTCGACCAGTCTAAGAACGGATATGCGGGCTACATAGACGGTGGAGCGAGTCAGGAGGAATTCTACAAGTTCATAAAGCCCATTCT
CGAGAAGATGGATGGTACGGAAGAACTGCTTGTGAAACTTAACAGAGAAGATCTTTTGCGGAAGCAGAGAACTTTCGACAACGGAAGTATACCACACCAGATACATCTCGGAGA
GCTTCATGCTATTCTCAGAAGACAAGAGGATTTCTACCCTTTCTTGAAGGATAACAGAGAAAAGATAGAGAAGATCCTCACGTTTAGGATCCCTTACTACGTAGGTCCTCTTGC
TCGCGGCAATAGTAGGTTCGCCTGGATGACCCGCAAGTCTGAAGAAACTATCACCCCTTGGAATTTCGAAGAGGTTGTAGACAAAGGTGCTTCAGCACAGAGTTTCATTGAGAG
GATGACCAACTTCGACAAGAACCTCCCCAACGAAAAGGTCCTGCCTAAGCACAGCCTCCTCTACGAATACTTTACTGTCTATAATGAGCTTACAAAAGTTAAGTACGTGACAGA
GGGAATGCGGAAGCCCGCATTCCTTTCCGGAGAACAAAAGAAGGCGATCGTGGATCTTCTCTTCAAGACGAACCGCAAGGTGACGGTTAAACAGTTGAAGGAAGATTACTTCAA
GAAGATAGAATGTTTTGATAGCGTGGAAATCAGCGGCGTCGAAGATAGGTTCAACGCTTCCCTGGGAACGTACCACGATCTCCTCAAGATTATCAAAGATAAGGACTTTCTTGA
TAACGAAGAGAATGAGGACATCTTGGAAGACATTGTTCTGACGCTCACCCTGTTCGAAGATCGCGAGATGATTGAGGAACGCTTGAAGACCTACGCACACCTGTTCGATGACAA
GGTTATGAAGCAACTTAAACGGCGCCGGTATACGGGCTGGGGACGGCTTTCGCGGAAGCTGATAAATGGAATCCGTGACAAGCAGTCTGGCAAGACAATACTCGACTTCTTGAA
GTCGGATGGTTTTGCCAATAGAAATTTTATGCAACTCATTCATGATGACTCGCTTACTTTTAAGGAGGACATCCAGAAGGCCCAGGTATCAGGACAGGGTGACTCTTTGCACGA
ACACATCGCGAACCTGGCGGGCTCCCCCGCGATTAAGAAGGGAATTTTGCAGACTGTCAAGGTGGTCGATGAACTCGTGAAGGTTATGGGACGTCATAAGCCGGAAAATATTGT
GATTGAGATGGCTCGCGAGAATCAAACAACACAGAAGGGCCAAAAGAACAGTAGAGAACGCATGAAGCGCATCGAAGAGGGCATCAAAGAGCTGGGCAGTCAGATCCTTAAAGA
ACATCCAGTCGAGAATACACAGCTTCAGAACGAAAAGCTGTACCTTTATTACCTTCAAAATGGGCGTGATATGTATGTGGATCAGGAACTCGATATCAATAGGCTGAGTGACTA
TGATGTCGACCATATCGTCCCGCAAAGTTTCCTCAAGGACGACAGTATAGACAACAAAGTTCTCACACGGTCAGATAAGAATCGCGGCAAGAGCGATAATGTACCGTCGGAGGA
GGTAGTCAAGAAGATGAAGAATTACTGGCGCCAGTTGCTCAACGCCAAGCTCATCACTCAGAGGAAATTTGACAACCTTACGAAAGCCGAGCGGGGCGGACTCTCTGAACTGGA
CAAGGCCGGTTTCATAAAGCGCCAGCTCGTTGAGACACGTCAAATTACTAAGCACGTCGCTCAAATATTGGATTCCCGCATGAATACTAAGTACGATGAGAATGATAAGCTCAT
ACGTGAAGTTAAGGTCATTACTCTCAAGTCCAAGCTTGTATCGGACTTCCGTAAGGACTTCCAATTCTACAAGGTCCGGGAAATCAATAATTATCACCATGCCCATGACGCTTA
TCTGAACGCGGTCGTGGGCACGGCACTCATTAAGAAATACCCAAAACTTGAGTCAGAATTTGTTTACGGGGACTATAAAGTTTATGACGTGCGGAAGATGATAGCGAAGTCGGA
ACAAGAGATAGGAAAGGCGACTGCAAAGTACTTTTTTTACTCCAACATAATGAATTTCTTTAAGACCGAAATAACCCTTGCAAACGGTGAAATCAGAAAGCGGCCTCTGATTGA
AACAAATGGCGAGACGGGCGAGATCGTCTGGGACAAGGGGAGGGACTTCGCAACGGTTCGCAAGGTCCTTAGCATGCCGCAAGTAAATATAGTTAAGAAGACGGAAGTTCAGAC
CGGCGGCTTTAGTAAAGAAAGCATACTTCCTAAAAGGAATTCCGACAAACTGATAGCGCGCAAGAAGGACTGGGATCCAAAGAAGTATGGAGGATTTGACTCCCCAACCGTTGC
TTATAGCGTGTTGGTAGTAGCCAAGGTGGAAAAGGGTAAGTCTAAGAAATTGAAGTCGGTGAAGGAGTTGTTGGGGATAACTATAATGGAGCGGAGTTCGTTCGAGAAGAACCC
AATTGACTTTCTCGAAGCCAAAGGCTACAAGGAGGTCAAGAAGGACCTGATTATTAAGTTGCCAAAGTACTCGCTCTTCGAACTCGAGAACGGGAGAAAGCGTATGCTGGCGTC
GGCGGGCGAGCTGCAGAAAGGAAACGAGCTGGCTTTGCCATCGAAATACGTAAATTTCCTGTACCTCGCCTCACATTATGAGAAGCTTAAAGGGTCTCCAGAAGACAATGAACA
GAAGCAGCTGTTTGTTGAACAGCACAAGCACTACTTGGACGAGATTATAGAACAAATCTCCGAGTTCTCTAAACGGGTTATCCTTGCAGACGCCAATTTGGATAAGGTCCTCTC
GGCTTATAATAAGCATAGAGATAAGCCAATCCGGGAGCAGGCTGAAAATATCATACACCTCTTTACGTTGACTAATTTGGGTGCGCCAGCGGCATTCAAGTACTTCGATACAAC
AATCGATCGTAAGCGCTACACAAGCACTAAGGAAGTCCTGGACGCGACGCTGATACACCAGTCCATTACTGGACTGTATGAAACCAGAATAGATCTTAGCCAGCTCGGCGGTGA

Pea 3A-ter

Ttgagagctccgatccaggcctcccagctttcgtccgtatcatcggtttcgacaacgttcgtcaagttcaatgcatcagtttcattgcccacacaccagaatcctactaagttt
gagtattatggcattggaaaagctgttttcttctatcatttgttctgcttgtaatttactgtgttctttcagtttttgttttcggacatcaaaatgcaaatggatggataagag
ttaataaatgatatggtccttttgttcattctcaaattattattatctgttgtttttactttaatgggttgaatttaagtaagaaaggaactaacagtgtgatattaaggtgca
atgttagacatataaaacagtctttcacctctctttggttatgtcttgaattggtttgtttcttcacttatctgtgtaatcaagtttactatgagtctatgatcaagtaattat

OsACT1-ter

gcaatcaagttaagtacagtataggctttggtaccttaGCCCCTAGGtaattcttcggacccaagaatgctaagccaagaggagctgttatcgccgtcctcctgcttgtttctc
tctttttgttgctgtttcttcattagcgtggacaaagttttcaaccggcctatctgttatcattttcttctattcaaagactgtaatacctattgctacctgtggttctcactt
gtgattttggacacatatgttcggtttattcaaatttaatcagatgcctgatgagggtaccagaaaaaatacgtgttctggttgtttttgagttgcgattattctatgaaatga
ataacatcgaagttatcatcccagtattttcgcatgaatgttcttttcttctgtcttgtgcatcagtgatctagtgcatgggagtttgtattgtgatgttcgacatcacgtaac
ttccactttgcctttgctgttcgatattttaatgacatgtcacacacacttctgatacttttctttcttggctattgtgccagcatgatgcaagatgcatcacagcatcagata
tattctcatcgtcaggctttagcagcacacgagcacgctttgccgcttaaaagttgtacggcgcagcttagacatcccctgtagaagtgataatcttttcacttttccttaaac
aaattgagaggggaaatggaaccatgtggatcagagaagcttttgtttctttacacaagaatatttggtacagtgggggtcctatgttcgtgggttcgtggcttggctgcctgt
cttcaaccaagtgttttcagttcaacatgttagcgtgtagaaagagcacaattctgtttatctccaaggtaaaatgtggcattctgttaaagaacatgatcctgccaattttt
aagtttcaatggaagaggaatgtaaagctttctatggtttgtgtacacaacacagtggaagaggagtgcaagctttctatggtttgtgtgcgcgttgtgtgtcagcacttcaat
tttgttagaaaatgaaagaaaaaaaaggatgatcatgcttatagtaaatcactctttttcctcgccttctgtacgttttgacttgacaagattttaaaatctgtacatgacctt
tgttttaaaattactttatgtatttccatctttcaagttatgcagatgtcatcacaaattgttacaccaatcaccaggctggctgtttatatattatcagaccaggctatatag
agtatactatactaactgttcatattatctggaaatcttgcttgctacttgagcggtaaaagggtatagatatgagggtcccc

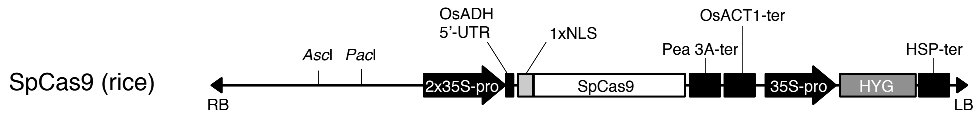

Supplemental Figure 6

SpCas9 expression binary vector for rice

backbone vector: pPZP200

gRNA expression vector for SaCas9

*Pac*I *AtU6-26* promoter

TTAATTAACTTTTTTTCTTCTTCTTCGTTCATACAGTTTTTTTTTGTTTATCAGCTTACATTTTCTTGAACCGTAGCTTTCGTTTTCTTCTTTTTAACTTTC

CATTCGGAGTTTTTGTATCTTGTTTCATAGTTTGTCCCAGGATTAGAATGATTAGGCATCGAACCTTCAAGAATTTGATTGAATAAAACATCTTCATTCTTA

AGATATGAAGATAATCTTCAAAAGGCCCCTGGGAATCTGAAAGAAGAGAAGCAGGCCCATTTATATGGGAAAGAACAATAGTATTTCTTATATAGGCCCATT

*Bbs*I *Bbs*I

TAAGTTGAAAACAATCTTCAAAAGTCCCACATCGCTTAGATAAGAAAACGAAGCTGAGTTTATATACAGCTAGAGTCGAAGTAGTGATTGggGTCTTCGAGA

scaffold for SaCas9

AGACCTGTTTTAGTACTCTGTAATTTTAGGTATGAGGTAGACGAAAATTGTACTTATACCTAAAATTACAGAATCTACTAAAACAAGGCAAAATGCCGTGTT

polyT *Asc*I

TATCTCGTCAACTTGTTGGCGAGATTTTTTTTCTAGACCCAGCTTTCTTGTACAAAGTTGGCATTAggcgcgcc

gRNA expression for SpCas9

*Pac*I *AtU6-26* promoter

TTAATTAACTTTTTTTCTTCTTCTTCGTTCATACAGTTTTTTTTTGTTTATCAGCTTACATTTTCTTGAACCGTAGCTTTCGTTTTCTTCTTTTTAACTTTC

CATTCGGAGTTTTTGTATCTTGTTTCATAGTTTGTCCCAGGATTAGAATGATTAGGCATCGAACCTTCAAGAATTTGATTGAATAAAACATCTTCATTCTTA

AGATATGAAGATAATCTTCAAAAGGCCCCTGGGAATCTGAAAGAAGAGAAGCAGGCCCATTTATATGGGAAAGAACAATAGTATTTCTTATATAGGCCCATT

*Bbs*I *Bbs*I

TAAGTTGAAAACAATCTTCAAAAGTCCCACATCGCTTAGATAAGAAAACGAAGCTGAGTTTATATACAGCTAGAGTCGAAGTAGTGATTGggGTCTTCGAGA

scaffold for SpCas9 polyT

AGACCTGTTTTAGAGCTAGAAATAGCAAGTTAAAATAAGGCTAGTCCGTTATCAACTTGAAAAAGTGGCACCGAGTCGGTGCTTTTTTTCTAGACCCAGCTT

*Asc*I

TCTTGTACAAAGTTGGCATTAggcgcgcc

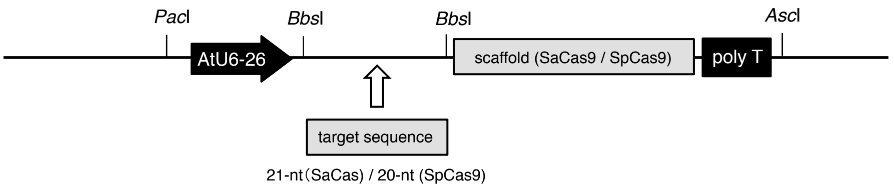

Supplementary Figure 7

The guide RNA expression vector
